# CRISPR-cas gene-editing as plausible treatment of neuromuscular and nucleotide-repeat-expansion diseases: A systematic review

**DOI:** 10.1371/journal.pone.0212198

**Published:** 2019-02-22

**Authors:** Haris Babačić, Aditi Mehta, Olivia Merkel, Benedikt Schoser

**Affiliations:** 1 Friedrich Baur Institute, Department of Neurology, Ludwig-Maximilians-University of Munich, Munich, Germany; 2 Faculty of Pharmacy, Ludwig-Maximilians-University of Munich, Munich, Germany; University of Florida, UNITED STATES

## Abstract

**Introduction:**

The system of clustered regularly interspaced short palindromic repeats (CRISPR) and CRISPR-associated proteins (cas) is a new technology that allows easier manipulation of the genome. Its potential to edit genes opened a new door in treatment development for incurable neurological monogenic diseases (NMGDs). The aim of this systematic review was to summarise the findings on the current development of CRISPR-cas for therapeutic purposes in the most frequent NMGDs and provide critical assessment.

**Methods and data acquisition:**

We searched the MEDLINE and EMBASE databases, looking for original studies on the use of CRISPR-cas to edit pathogenic variants in models of the most frequent NMGDs, until end of 2017. We included all the studies that met the following criteria: 1. Peer-reviewed study report with explicitly described experimental designs; 2. *In vitro*, *ex vivo*, or *in vivo* study using human or other animal biological systems (including cells, tissues, organs, organisms); 3. focusing on CRISPR as the gene-editing method of choice; and 5. featured at least one NMGD.

**Results:**

We obtained 404 papers from MEDLINE and 513 from EMBASE. After removing the duplicates, we screened 490 papers by title and abstract and assessed them for eligibility. After reading 50 full-text papers, we finally selected 42 for the review.

**Discussion:**

Here we give a systematic summary on the preclinical development of CRISPR-cas for therapeutic purposes in NMGDs. Furthermore, we address the clinical interpretability of the findings, giving a comprehensive overview of the current state of the art. Duchenne’s muscular dystrophy (DMD) paves the way forward, with 26 out of 42 studies reporting different strategies on *DMD* gene editing in different models of the disease. Most of the strategies aimed for permanent exon skipping by deletion with CRISPR-cas. Successful silencing of the *mHTT* gene with CRISPR-cas led to successful reversal of the neurotoxic effects in the striatum of mouse models of Huntington’s disease. Many other strategies have been explored, including epigenetic regulation of gene expression, in cellular and animal models of: myotonic dystrophy, Fraxile X syndrome, ataxias, and other less frequent dystrophies.

Still, before even considering the clinical application of CRISPR-cas, three major bottlenecks need to be addressed: efficacy, safety, and delivery of the systems. This requires a collaborative approach in the research community, while having ethical considerations in mind.

## Introduction

Genome editing has been a hot topic in philosophy and science long before decoding the human genome. There have been major successes in developing different approaches to genome editing, hoping to implement it for therapeutic purposes.

In principle, editing a sequence in the genome requires two steps: successful and specific recognition by a “DNA-binding domain”, and an “effector domain” to cleave the DNA or regulate transcription [1]. Inducing a double-stranded break (DSB) in the DNA shows a higher rate of gene modification than not inducing one, mainly through activating one of the two DNA repair mechanisms: non-homologous end joining (NHEJ) or homology directed repair (HDR) [2–6]. NHEJ introduces insertions or deletions within a sequence, whereas HDR requires a donor sequence that through recombination with the targeting sequence can lead to point mutations or insertions [7].

Endonucleases, specifically meganucleases, have been studied and manipulated for this purpose, motivating scientists to push forward. This led to the development of other bioengineering tools in genome editing, such as zinc-finger nucleases (ZFNs) and transcription activator-like effector nucleases (TALENs). Both have a DNA-binding domain attached to the FokI nuclease domain, which acts as an effector domain[8, 9]. However, these approaches, although successful, require extensive engineering of new proteins for each new editing site, making it a long, highly specialised, and expensive process [1]. None of these genome-editing tools have sparked such interest in the scientific community as did the discovery of the Clustered Regularly Interspaced Short Palindromic Repeats (CRISPR) and CRISPR-associated protein (cas) systems[10].

### CRISPR-cas in nature

CRISPR-cas systems are part of the immune system of bacteria and Archaea, used to defend themselves from bacteriophages [11–13]. After they survive an attack, many bacteria and Archaea store protospacers (parts of the foreign DNA of their invaders—the bacteriophages or plasmids) into the CRISPR gene loci. These CRISPR sequences along with the protospacers serve as a cellular immune memory[14–16]. When transcribed and cleaved into mature RNA, they recognise and bind the DNA sequences of the invaders, guiding the effector domain—one of the many cas protein endonucleases—to specifically recognise and cleave the genome of the invader, thereby fighting infection[1, 7].

### CRISPR-cas as a genome-editing tool

**Fig 1:**
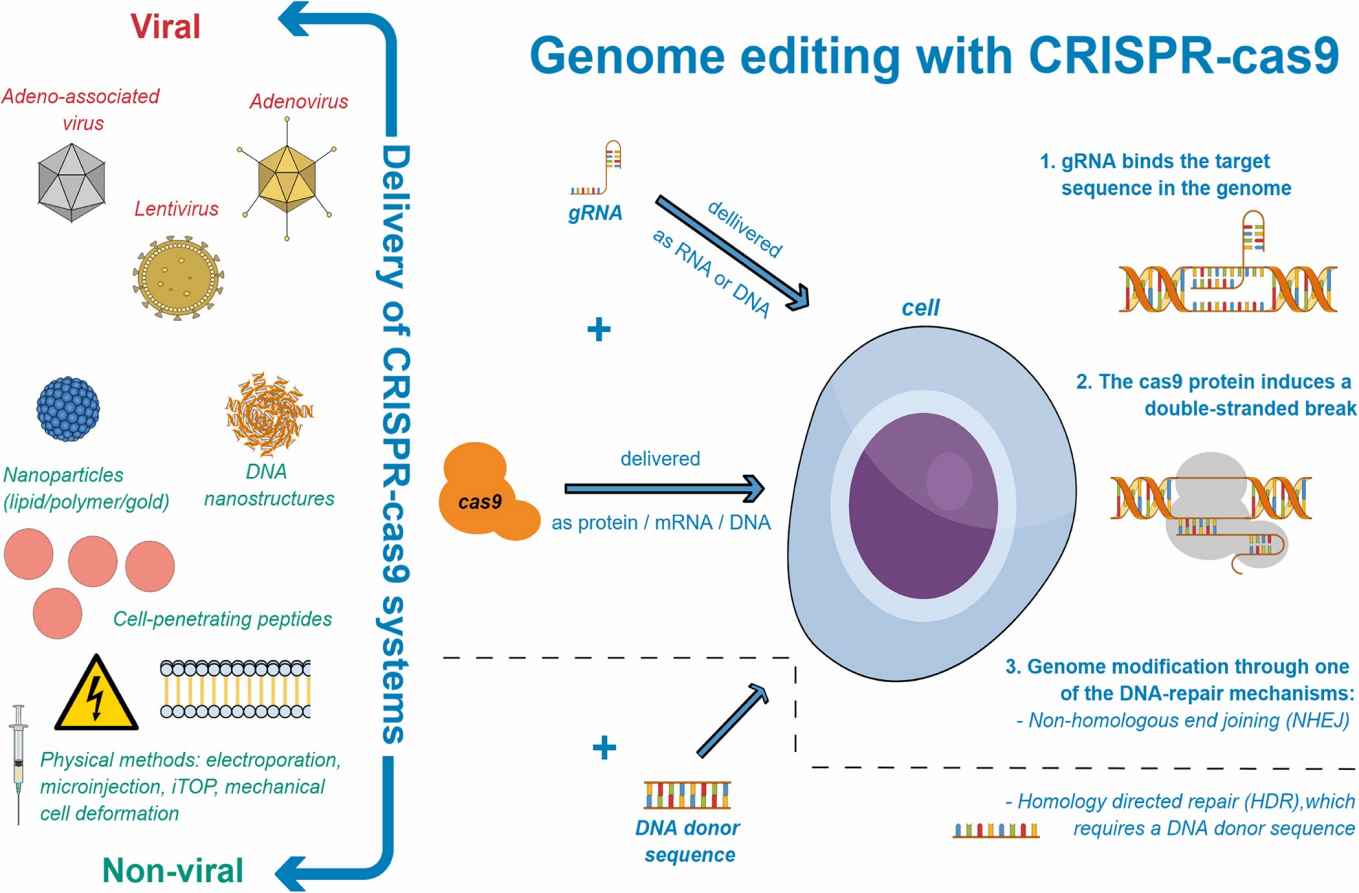
Genome editing with CRISPR-cas9. **On the right—schematic depiction of genome-editing with CRISPR-cas.** After delivering the CRISPR components, during the G1/S phase of the cell cycle: 1. The gRNA (DNA-binding-domain) binds the target sequence within the genome; 2. This gRNA-DNA complex is specifically recognised by the cas9 protein (the effector domain), which induces a double-stranded break in the DNA; 3. Genome modification occurs, mainly through activating one of the two DNA repair mechanisms: non-homologous end joining (NHEJ) or homology directed repair (HDR). NHEJ introduces insertions or deletions within a sequence, whereas HDR requires delivery of a donor sequence that through recombination with the targeting sequence can lead to point mutations or insertions. On the left–different delivery methods are used to deliver the CRISPR-cas9 components: viral and non-viral. Viral methods are usually more efficient but raise concerns such as immunogenicity.

The potential of this discovery kindled the development of a new gene-editing tool. It demonstrated a natural system that can specifically and efficiently cleave DNA, while carrying the DNA-binding and specificity domain (in a form of RNA) separate from the effector domain (the cas protein)[17, 18]. This led to the adaptation of the CRISPR-cas systems for genome editing (Fig 1). Different research groups, using the CRISPR-cas system of the bacteria *Streptococcus pyogenes*, engineered a version of an RNA molecule, named guide RNA (gRNA)[19, 20]. The gRNA provides target specificity by binding to its complementary DNA sequence, resulting in a DNA-RNA hybrid. The gRNA is easy to adapt and targets various loci within the genome. It is specifically recognised by the cas9—one of the many cas proteins, found in *S*. *pyogenes* and other bacteria. The cas9 is an endonuclease that requires a G-rich protospacer adjacent motif (PAM) sequence to target the DNA. In other words, usually, but not necessarily, the 2–6 base pair DNA sequence (PAM) that follows the targeted sequence should be 5'-NGG-3’ (any nucleotide followed by two guanine nucleobases) in order to be recognised by cas9. Once cas9 recognises the gRNA-DNA binding it cuts proximally to the recognition site, leaving blunt ends of the DSB [1, 21].

Other cas9 endonucleases have been engineered to recognise different PAMs. Cas9 can be also catalytically inactivated (dCas9) by mutations in the endonuclease domain. This dCas9 can still bind to the sgRNA and thereby be directed to different loci in the genome. By binding to the DNA, dCas9 prevents transcription factors and other molecules from accessing the sequence, thereby preventing transcription of that gene to mRNA and subsequent translation to protein. Furthermore, dCas9 can be modified with mutations that allow it to bind to other transcription modulators and result in epigenetic activation/silencing [22].

Other cas proteins have been bioengineered to comprise different properties. A new cas protein–cpf1 (CRISPR from *Prevotella* and *Francisella* 1, also known as cas12a) has been adapted for genome editing. It is different from cas9 in four aspects: 1. it requires one RNA molecule (crRNA) for making the gRNA whereas cas9 requires two (crRNA and trRNA); 2. it cuts distally from the recognition site, whereas cas9 cuts proximally; 3. it leaves sticky ends, whereas cas9 leaves blunt ends after cutting; and 4. it targets a T-rich PAM, whereas cas9 targets a G-rich PAM[23].

With CRISPR-cas, for the first time, the DNA-binding domain was separate from the effector domain, allowing an easier manipulation and adjustment of the DNA-binding domain to the targeted sequence. In addition, it is simpler to use, compared to other gene editing tools, much less expensive, and does not require very sophisticated technology—making it easy to implement in almost any modern lab.

The discovery of CRISPR-cas revolutionised the field of genome editing. The overall number of publications on genome editing rose rapidly in the last couple of years. Approximately 90% of around 2500 publications in 2016 were on CRISPR-cas as a method used to edit the genome of bacterial, insectoid or mammalian cells [10].

### Delivery of CRISPR-cas systems

Many different approaches have been developed to deliver CRISPR-cas systems to cells. Primarily, the two necessary domains of CRISPR–the gRNA and the cas9 protein—are deliverable in different forms. The gRNA can be delivered as DNA cloned into a plasmid or as RNA. The cas9 protein however, can be delivered as plasmid DNA, *in vitro* transcribed mRNA or as protein. Delivery of the ribo-protein complex, prepared at the bench, is advantageous because the cas9 protein remains inside the cells for a short period, resulting in minimal off-target effects [1].

Secondary, aside from physical methods (such as electroporation, microinjection, induced transduction by osmocytosis and propanebetaine (iTOP), and mechanical cell deformation), two different types of carriers can be used to deliver these different forms of gRNA and cas9: viral and non-viral. We refer to them here as “delivery systems”. Viral delivery is available by lentiviruses (LV), adenoviruses (AdV), and adeno-associated viruses (AAV). Non-viral delivery is possible by: lipid nanoparticles, polymer nanoparticles, cell-penetrating peptides (CPP), DNA nanostructures, and gold nanoparticles. All of them have their own advantages and disadvantages, discussed in detail by Liu *et al*. (2017)[24]. Both viral and non-viral carriers entrap or entail the different forms of gRNA and/or cas protein, protecting them from degradation. This makes the delivery more stable and efficient. Furthermore, they provide the opportunity to control drug release, improving the pharmacokinetics and bioavailability of the drugs.

### CRISPR-cas as plausible causal treatment of the most frequent monogenic neurological diseases

Successful, precise and safe genome editing has the potential to change our approach in research, industry, and medicine. In the last six years, many research groups have focused on manipulating, adapting, and improving the methodology of CRISPR-cas genome editing, with the hope to use it in clinical application someday. CRISPR-cas seems a promising strategy for treatment of monogenic neurological diseases (NMGDs) as well. There is one underlying causal event in all of them—a pathogenic variant, which leads to subsequent dysfunction and cell death. Currently, these diseases are treatable to some extent (symptom management), but not curable. Developing an approach to remove or replace the mutated genes could provide a new causal treatment for NMGDs, by correcting the mutation and stopping the subsequent pathophysiological mechanisms. A possible complication can appear due to the fact that gRNAs are usually of around 20 bp of size; there is a concern that the gRNA might recognise different targeting sequences within the genome, leading to non-targeted DSBs and “off-target” gene editing effects, when administered at high concentrations. Another question of practical significance is—if successful, how best to deliver these systems to gain highest efficiency with lowest safety concerns.

The plethora of publications makes it worthwhile to follow and understand the current state of the development on the use of CRISPR-cas for therapeutic purposes in neurology. With this systematic review we summarise the state of development on the use of CRISPR-cas for therapeutic purposes in the most frequent NMGDs up until end of 2017. Furthermore, we address the challenges in the pre-clinical development of this biological therapy, as well as its opportunity for translation to the bedside.

## Methods and data acquisition

We have searched the MEDLINE and EMBASE databases. Studies of interest were original, experimental work on development of CRISPR-cas systems for therapeutic purposes, to edit gene mutations that cause NMGDs.

All following inclusion criteria were to be met: We included all the studies that met the following criteria: 1. Peer-reviewed study report with explicitly described experimental designs; 2. *In vitro*, *ex vivo*, or *in vivo* study using human or other animal biological systems (including cells, tissues, organs, organisms); 3. focusing on CRISPR as the gene-editing method of choice; and 5. featured at least one NMGD. The specified diseases within the search keywords are the most frequent NMGDs.

After defining the keywords based on the inclusion criteria, we additionally searched for the corresponding Mesh terms in PUBMED, as well as the corresponding Emtree terms in EMBASE. All keywords used for the search in both databases can be found in S1 Table. On 17^th^ December 2017, we downloaded and merged all the identified references with abstracts to EndNote X8. We used neither language nor time restriction. Using the EndNote function “Find duplicates”, we identified and removed the duplicates automatically. Two reviewers screened the papers based on title and abstract, individually and independently, and excluded reviews, notes, comments, and conference abstracts manually. The papers that met the criteria or had no clear exclusion criteria were included for further processing. Finally, after reading the full papers, we selected the eligible studies for qualitative synthesis.

Up to this date, we did not find a valid bias assessment tool applicable to experimental studies on CRISPR-cas. We did not assess the studies for bias, to avoid invalid conclusions. We followed the recommendations on Preferred Reporting Items for Systematic Reviews and Meta-Analyses (PRISMA), expressed in the PRISMA Statement[25] (S1 Checklist).

## Results

**Fig 2:**
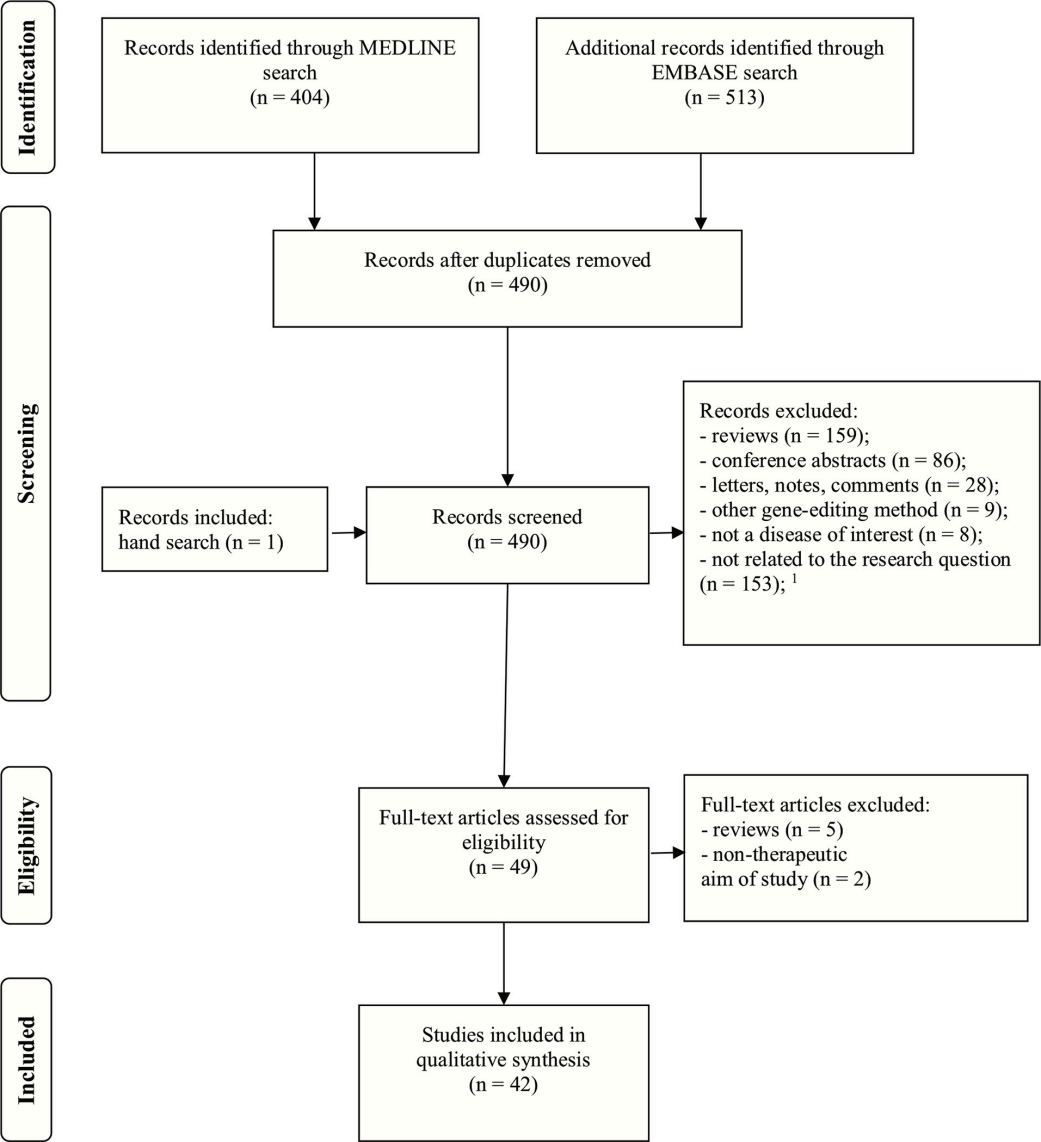
PRISMA flow diagram for the systematic review.

**Fig 3:**
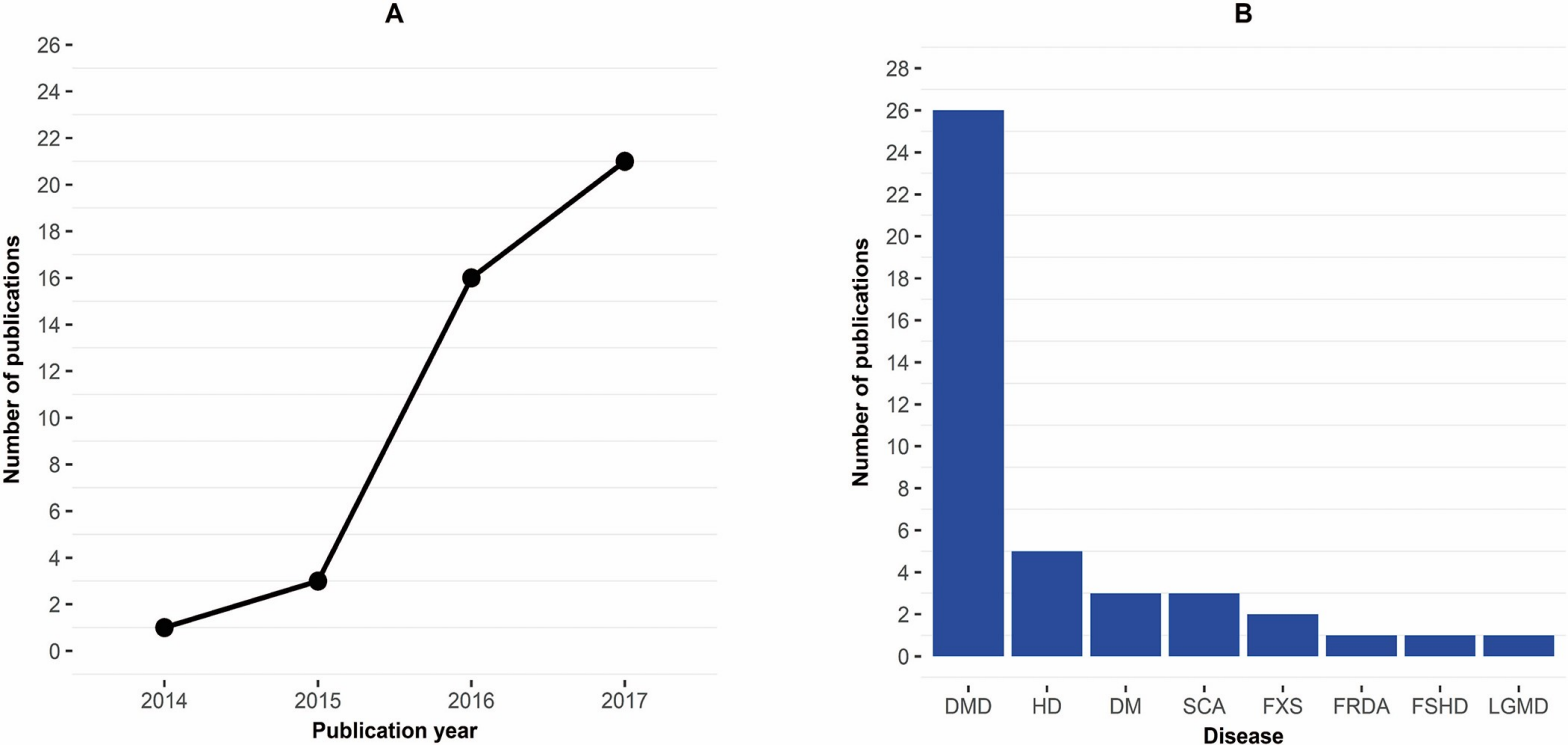
**Number of CRISPR publications: A. per year; B. per disease type.** Abbreviations: DMD–Duchenne’s muscular dystrophy, HD–Huntington’s disease, DM–myotonic dystrophy, SCA–spinocerebellar ataxia, FXS–fragile X syndrome, FRDA–Friedreich’s ataxia, FSHD–facioscapulohumeral dystrophy, LGMD–limb-girdle muscular dystrophy.

Fig 2 shows the total number of references obtained from MEDLINE and EMBASE, as well as the numbers of articles screened, assessed for eligibility, and finally selected for the review. Fig 3 depicts the number of publications per year and disease type. All conference abstracts (n = 86) originated exclusively from EMBASE, and none were detected with PUBMED. Of the 42 selected papers, 34 emerged from both databases, 7 only from MEDLINE and we included one paper detected by manual search after screening (concluded by 31. December 2017). Tables 1 and 2 give an overview of the selected studies. Tables 3 and 4 provide a detailed overview on CRISPR-cas editing strategies, efficiency, off-target and outcome assessment, for the *in vivo* studies included in this review.

**Table 1:** Overview of the studies on Duchenne’s muscular dystrophy included in the systematic review.

Authors	Year	Disease	Origin of model	Delivery to cells	Key-point(s):
Amoasi *et al*. [39]	2017	DMD*†	*ΔEx50* M	AAV 9	New mouse model: *ΔEx50*. Early delivery increased editing-efficiency of exon 51
Bengtsson *et al*. [59]	2017	DMD*†	*mdx*^*4cv*^ M	EP, AAV 6	Excised exon 52&53. Dual-, compared to single-, vector delivery was more efficient
Duchene *et al*. [60]	2018	DMD*	H	LF 2000	Protocol for *DMD* correction, using the CinDel Method
Ehrke-Schulz *et al*. [57]	2017	DMD*	H	HC-AdV	Designed a new viral system (HC-Adv), with a large delivering capacity (35 kb)
El Refaey *et al*. [49]	2017	DMD*†	*mdx/Utr*^*+/−*^ M	EP, AAV rh74	Demonstrated functional improvement in cardiac contractility (papillary muscle)
Iyombe-Engembe *et al*. [61]	2016	DMD*†	H, hDMD/*mdx* M	LF 2000, EP	Deleted exons 51–53, creating a hybrid exon 50–54 in mice that contain human *DMD*
Kyrychenko *et al*. [46]	2017	DMD*	H	NF	Editing the ABD-1 domain showed improvement in functionality of cardiomyocytes
Lattanzi *et al*. [45]	2017	DMD*	H	LV	Demonstrated a strategy for editing the exon 2 duplication using one gRNA
Lee *et al*. [64]	2017	DMD*†	*mdx* M	CGNP	Induced HDR, delivering the CRISPR components with new Gold-nanoparticles.
Li *et al*. [56]	2015	DMD*	H	EP	Exon 44 skipping, frameshifting, and knockin. Knockin restored full protein length
Liao *et al*. [66]	2017	DMD†	*Cas9/mdx*, *mdx* M	AAV 9	Epigenetically up-regulated expression of utrophin, using dead cas9 (dCas9-VP160)
Long *et al*. [65]	2014	DMD†	*mdx* M	MI	Germline editing in mice produced mosaic animals with 2–100% *DMD* correction
Long *et al*. [50]	2016	DMD†	*mdx* M	AAV 9	Different modes of AAV 9 delivery show varying efficiency in restoring dystrophin
Maggio *et al*. [62]	2016	DMD*	H	AdV	Excised a large region encompassing exons 44–54, covering many mutations
Maggio *et al*. [58]	2016	DMD*	H	AdV	Explored editing strategies, using CRISPR-cas alone and combined with TALENs
Mou *et al*. [52]	2017	DMD*	M	LV	Used single gRNA skipping of exon 23 in C2C12 mouse cells.
Nelson *et al*. [53]	2016	DMD†	*mdx* M	AAV 8	Deletion of exon 23 improved muscle function in both adult and neonatal mice
Ousterout *et al*. [44]	2015	DMD* ‡	H in M	LF & EP	Edited patient myoblasts, engrafted in immunodeficient mice, restored dystrophin
Perrin *et al*. [67]	2017	DMD*†	*Rag/mdx* M	TransfeX, LF & EP	Increased the expression of Laminin subunit α1, using dCas9-VP160
Tabebordbar *et al*. [54]	2016	DMD*†	*mdx* M	AAV 9	Gene modifications possible in terminally differentiated muscle cells
Wojtal *et al*. [47]	2016	DMD*	H	EP	Increased the expression of utrophin, using dCas9-VP160
Xu *et al*. [48]	2016	DMD*†	*mdx* M	EP, AdV	Deletion of exons 21–23 improved sarcolemal integrity in skeletal muscle cells
Young *et al*. [63]	2016	DMD* ‡	H in NSG-*mdx* M	NF	Deletion of 725 kb, encompassing exons 45–55, restored dystrophin in muscle cells
Zhang *et al*. [23]	2017	DMD*†	H, mdx M	NF and MI	Used a new endonuclease (cpf1) for *DMD* editing in human iPSCs and mice
Zhu *et al*. [55]	2017	DMD*‡	*mdx* M	LF 3000, AdV	Developed a fibrin gel to propagate CRISPR-cas9-corrected muscle stem cells

* Concept proven *in vitro*.

† Concept proven *in vivo*.

‡ Concept proven *ex vivo*.

**Abbreviations:** H–human, M–mouse, *mdx–*mouse model of DMD, AdV–Adenovirus, AAV–Adeno-associated virus, LF–Lipofectamine, PEI–Polyethylenimine, CGBP–CRISPR-Gold nanoparticles, LF–Lipofectamine, EP–Electroporation, NF–Nucleofection, HC-AdV–High-capacity adenoviral vectors, MI–Microinjection.

**Table 2:** Overview of the studies on other monogenic neurological diseases included in the systematic review.

Authors	Year	Disease	Origin of model	Delivery to cells	Key-point(s):
Kolli *et al*. [110]	2017	HD*	*YAC128* M	LV	Excised *mHTT* exon 1 in mesenchymal stem cells extracted from mice
Monteys *et al*. [112]	2017	HD*†	H, *BacHD* M	LF, EP, AAV	Reduced human *mHTT* levels in striata of treated right brain hemispheres of mice
Shin *et al*. [109]	2016	HD*	H	EP	Patient-specific inactivation of the mutant haplotype in patient-derived cells.
Xu *et al*. [111]	2017	HD*	H	NF	Editing of the *mHTT* lead to reversal of the phenotypic abnormalities in iPSCs
Yang *et al*. [113]	2017	HD*†	*HD140Q-KI* M	AAV	Treated 9-month old mice had reversal of neuropathology and behavioural changes
Marthaler *et al*. [122]	2016	SCA2*	H	NF	Replaced the CAG-expanded *ATXN2* gene with a normal-sized in iPSC line H271
Marthaler *et al*. [123]	2016	SCA2*	H	NF	Correction of another model of SCA2—iPSC line H195
Marthaler *et al*. [124]	2016	SCA2*	H	NF	Correction of another model of SCA2 –iPSC line H266
Ouellet *et al*. [121]	2017	FRDA*†	*YG8R*, *YG8sR* M	LF, EP	Removal of repeats expansion reduced *FXN* levels in treated YG8R-fibroblasts
Park *et al*. [116]	2015	FXS*	H	EP	Single gRNA-targeting of the CGG repeat led to excision of the mutation in iPSCs
Xie *et al*. [117]	2016	FXS*	H	LF 3000, NF	Dual cleavage of the CGG repeats within the *FMR1* gene reactivated the *FMR1* gene
Turan *et al*. [99]	2016	LGMD*	H	NF	CRISPR-cas9-ssODN-mediated HDR of point mutations in LGMD2B & LGMD2D
Himeda *et al*. [102]	2016	FSHMD*	H	LV	Used dCas9 fused to transcriptional effectors for *DUX4* epigenetic downregulation
Kemaladewi *et al*. [104]	2017	MDC1A*†	*dy*^*2J*^*/dy*^*2J*^ M	AAV 9	*LAMA2*-editing restored Laminin2 and improved muscle strength in treated mice
van Agtmaal *et al*. [94]	2017	DM1*	H, M	NF	Excision of the expanded *DMPK* gene cleared cells from ribunuclear foci
Pinto *et al*. [96]	2017	DM1 *†	H, HSALR M	AAV	dCas9 regulation of mRNA^exp^ transcription improved myotonia in treated mice
Batra *et al*. [95]	2017	DM1*	H, Mnk	LF 3000, LV	dCas9-cleavage of mRNA^exp^ removed ribonuclear foci and corrected mis-splicing

* Concept proven *in vitro*

† Concept proven *in vivo*

**Abbreviations:** H–human, M–mouse, Mnk–African green monkey, *YAC128*, *BacHD*, HD140Q-KI–mouse models of HD, *YG8R*, *YG8sR*–mouse models of FRDA, *dy*^*2J*^*/dy*^*2J*^ –mouse model of MDC1A, *HSA*^*LR*^–mouse model of DM1, AAV–Adeno-associated virus, LF–Lipofectamine, EP–Electroporation, NF–Nucleofection, LV–Lentivirus.

**Table 3:** Overview on CRISPR-cas editing strategies and outcome assessment for *in vivo* studies in animal models.

Authors	Disease	Route of administration	Editing Strategy	Off-target assessment	Off-target events (OTEs)	Outcome RNA assessment†	Outcome Protein assessment†	Improved functional outcomes
Amoasi *et al*. (2017) [39]	DMD^aav^	I.M., I.P.	sgRNA, cas9	T7E1 assay, deep sequencing	no OTEs in top 6 predicted loci	n.r.	WB, IHC	histopathology, grip strength test.
Bengtsson *et al*. (2017) [59]	DMD^aav^	I.M., R.O.	single vs. two gRNAs, cas9	deep sequencing	<1% in top 5 predicted loci	RT-PCR	WB, IHC	localization of nNOS, muscle force generation.
El-Refaey *et al*. (2017) [49]	DMD^aav^	R.O., I.P., I.V.	two gRNAs, cas9	n.r.	n.r.	RT-PCR	WB, IHC	fibrosis reduction, contractility of papillary muscles.
Liao *et al*. (2017) [66]	DMD^aav^	I.M.	sgRNA, dead cas9	n.r.	n.r.	qRT-PCR	IHC	muscle mass, grip strength test.
Long *et al*. (2016) [50]	DMD^aav^	I.M., R.O., I.P.	two gRNAs, cas9	T7E1 assay	no OTEs in top 10 predicted loci	RT-PCR	WB, IHC	histopathology, serum CK, grip strength test.
Iyombe-Engembe *et al*. (2016) [61]	DMD^ep^	I.M.	two gRNAs, cas9	n.r.	n.r.	n.r.	WB	n.r.
Lee *et al*. (2017) [64]	DMD^np^	I.M.	sgRNA, cas9, template (HDR)	deep sequencing	0.005–0.2% in top predicted loci	n.r.	WB, IHC	histopathology, four-limb hanging test.
Nelson *et al*. (2016) [53]	DMD^aav^	I.M, I.P., I.V.	two gRNAs, cas9	deep sequencing	< = 1%	RT-PCR, ddPCR	WB, IHC	localization of nNOS, muscle force, resistance to muscle damage.
Tabebordbar et al. (2016) [54]	DMD^aav^	I.M, I.P.	two gRNAs, cas9	next-generation sequencing	<0.1% in top 8 predicted loci	RT-PCR, Taqman-RT-PCR	WB, capillary immunoassay, IHC	histopathology, muscle force generation.
Xu et al. (2016) [48]	DMD^adv^	I.M.	two gRNAs, cas9	n.r.	n.r.	qRT-PCR	WB, IHC	sarcolemmal proteins’ localization, myofiber damage after stress.
Zhang et al. (2017) [23]	DMD^mi^	C&Pn	single vs. two gRNAs, cpf1 (germline)	T7E1 assay, capillary electrophoresis	no OTEs in top 10 predicted loci	RT-PCR	WB, IHC	serum CK, grip strength test.
Long *et al*. (2014) [65]	DMD^mi^	C&Pn	sgRNA, cas9, template (HDR, germline)	deep sequencing	<2.5% in top 10 predicted loci	n.r.	WB, IHC	Histopathology, serum CK, grip strength test.

^aav^—adeno-associated virus

^adv^–adenovirus

^ep^–electroporation

^np^–nanoparticles

^mi^–microinjection.

† Primary outcome of interest for DMD in these studies was increased expression of dystrophin.

**Abbreviations:** I.M.—intramuscular, I.P.—intraperitoneal, R.O.—retroorbital, I.V.–intravenous, C&Pn–cytoplasmic and pronuclear, nNOS—neuronal nitric oxide synthase, CK—creatine kinase, IHC—immunohistochemistry, WB—Western blot, RT-PCR—real-time polymerase chain reaction, qRT-PCR—quantitative RT-PCR, IF–immunofluorescence, n.r.—not reported, sgRNA–single gRNA

**Table 4:** Overview on CRISPR-cas editing strategies, off-target and outcome assessment for *in vivo* studies in animal models (continued).

Authors	Disease	Route of administration	Editing Strategy	Off-target assessment	Off-target events (OTEs)	Outcome RNA assessment†	Outcome Protein assessment†	Improved functional outcomes
Perrin et al. (2017) [67]	DMD^ep^	I.M.	sgRNA, dead cas9	qRT-PCR	no OTEs in top 8 predicted loci	qRT-PCR	WB, IHC	Lama1 gene expression induction.
Monteys *et al*. (2017) [52]	HD^aav^	injected in one striatum side	sgRNA, cas9	Sanger sequencing	no OTEs in top 11 predicted loci	qRT-PCR	WB	n.r.
Yang et al. (2017) [113]	HD^aav^	injected in both striatum sides	two gRNAs, cas9	whole genome sequencing	rare OTEs	n.r.	WB, IHC	histopathology, rotarod and balance beam test, grip strength test.
Ouellet et al. (2017) [121]	FRDA^ep^	I.M.	two gRNAs, cas9	n.r.	n.r.	qRT-PCR	WB	n.r.
Kemaladewi et al. (2017) [104]	MDC1A^aav^	I.M., I.P, I.V.	two gRNAs, cas9	T7E1 assay	no OTEs in top 20 predicted loci	RT–PCR, qPCR, ddPCR	WB, IHC	histopathology, open-field activity test, muscle force generation,
Pinto et al. (2017) [96]	DM1^aav^	I.V.	sgRNA, dead cas9	RNA sequencing	negligible	FISH	WB, IF	ribonuclear foci, myotonia (electromyography assessment)

^aav^—adeno-associated virus

^ep^–electroporation.

† Primary outcomes of interest for HD, FRDA, MDC1A, and DM1 were decreased expression of huntingtin, restoration of frataxin, restoration of Lama2 protein, and reduction in RNA foci, respectively.

**Abbreviations:** I.M.—intramuscular, I.P.—intraperitoneal, I.V.–intravenous, IHC—immunohistochemistry, WB—Western blot, RT-PCR—real-time polymerase chain reaction, qRT-PCR—quantitative RT-PCR, FISH- fluorescent in situ hybridisation, IF–immunofluorescence, ddPCR—digital drop PCR, n.r.—not reported, sgRNA–single gRNA

## Discussion

The knowledge on developing therapeutic CRISPR-cas gene-editing strategies in NMGDs is steadily growing. Starting with the first publication by Long *et al*. in 2014, the number of publications grew rapidly (Fig 3). Among the NMGDs, Duchenne’s muscular dystrophy (DMD) is the front-runner in the preclinical development of CRISPR-cas systems for therapeutic purposes. Based on to the current pace of development DMD is likely to be the first one to reach clinical phase. The therapeutic use of CRISPR-cas has three bottlenecks: efficacy, safety, and delivery of CRISPR-cas systems. These need to be addressed in detail, in order to properly assess and weigh the benefits over the risks, before implementing CRISPR-cas in clinical trials. Here, we give an overview of the current development, addressing each disease separately, focusing on results that we consider of highest relevance in translation to the clinic.

### Duchenne’s muscular dystrophy (DMD)

DMD is the most prevalent, severe muscular dystrophy that affects 1 in 3,500–5,000 boys. [26] Usually diagnosed at the age of 3 to 5 years old, it presents with truncal and proximal lower-limb muscle weakness. Boys affected by DMD have motor delay, abnormal gait, and difficulty rising from the ground. Disease progression leads to upper-limb and distal muscle weakness. Eventually, it affects the respiratory muscles, leading to chronic respiratory insufficiency. Beside skeletal muscle involvement, weakness affects the heart muscle, leading to dilated cardiomyopathy. By the age of 12, most boys are bound to the wheelchair. [27] In the past, DMD patients were dying usually in their teenage and adolescent years. Nowadays life expectancy reaches up to 40 years of age, probably due to improved respiratory and cardiac management. Still, most of the patients die around 30 years of age. [28]

#### Pathogenesis

DMD is an X-linked recessive disease, caused by mutations in the dystrophin gene (*DMD*). [29, 30] Located on the short (p) arm of the X chromosome (Xp21 locus), containing 79 exons encompassing 2.3 x 10^6^ base pairs (bp), *DMD* is the largest gene in the human genome. [31, 32] The dystrophin protein has a rod-shaped structure and consists of four domains: the first actin-binding domain (ABD-1), the central rod domain (which includes the second actin-binding domain–ABD-2), the cysteine-rich domain, and the carboxyl-terminal domain. Expressed in skeletal and cardiac muscle cells, dystrophin proteins physically connect the actin filaments in the sarcomere with the dystroglycans in the sarcolemma. [33, 34] This coupling provides stability to the sarcolemma as dystroglycans bind the laminin-2 proteins within the extracellular matrix. Furthermore, the dystrophin protein interacts, directly or indirectly, with many dystrophin-glycoprotein components: synemin/desmulin, syncoilin, desmin, filamin, plectin, vinculin, aquaporin-4, caveolin-3, neuronal nitric oxide synthase (nNOS), and others. All these form protein assemblies termed costameres, which function as absorbers (“cushions”) of mechanical stress in the myofibres. [35, 36] Absence of dystrophin in DMD leads to increased mechanical stress in the muscle cells during movements and physical exercise, causing tearing of the sarcolemma, necrosis, inflammation, and subsequent fibrosis. [34, 37, 38]

Presumably due to its large size, the *DMD* gene is more susceptible to mutations than other genes. More than 4,000 mutations have been identified, with one-third appearing *de novo*. [39] Approximately two-thirds of *DMD* mutations are large deletions, followed by duplications, representing 10% of all mutations. These are usually clustered at “hotspot” regions: between exons 2–20 and exons 44–53. The remaining are small deletions or point mutations. [40] Overall, the majority of *DMD* mutations that disrupt the reading frame of the dystrophin protein (out-of-frame mutations) lead to prematurely truncated, unstable, and dysfunctional proteins, which are degraded in the muscle cells. On the other hand, *DMD* mutations that maintain the reading frame (in-frame mutations) result in internally deleted, shorter but still functional dystrophin. This shorter dystrophin, with lower molecular weight, leads to a milder form of dystrophinopathy, called Becker muscular dystrophy (BMD). [27] BMD has lower prevalence (1 in 11,500 men), later onset and a varying presentation. It usually manifests with waddling gait, skeletal and respiratory muscle weakness, and cardiomyopathy. BMD patients have longer life expectancy. [28]

#### CRISPR-cas editing strategies

**Fig 4:**
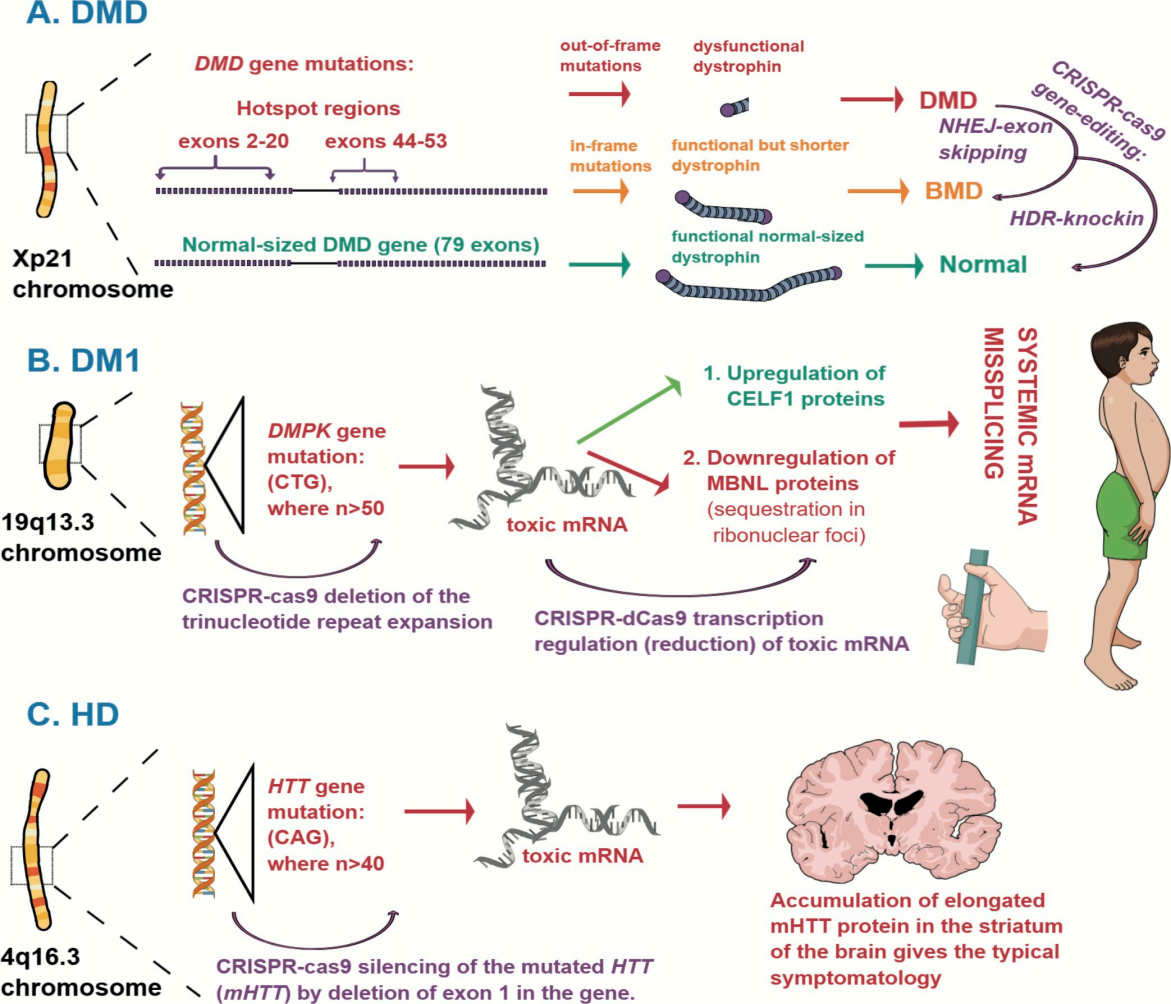
Pathophysiology of the most frequent NMGDs and CRISPR-cas strategies for treatment. A. Duchenne’s Muscular Dystrophy (DMD) is caused by mutations in the *DMD* gene, located on chromosome X. Mutations which lead to the formation of dysfunctional dystrophin causes the typical childhood-onset disease with severe muscular weakness and wasting, leading to death in the adolescent age. Mutations that allow the expression of a functional but shorter dystrophin cause a less severe phenotype of the disease known as Becker’s Muscular Dystrophy (BMD). Two CRISPR-cas9 strategies have been explored so far: 1. deletion of exons where the DMD mutation is located, which leads to BMD-like dystrophin expression and phenotype in mice; 2. HDR-mediated knockin that has low efficiency. B. Myotonic Dystrophy type 1 (DM1) is caused by a CTG nucleotide repeat expansion in the *DMPK* gene. An elongated transcribed mRNA shows toxic effects in the cells due to mis-splicing of proteins, which leads to myotonia, muscular wasting (see a schematic depiction of a typical phenotype in a boy with congenital DM1), and endocrine disorders. Authors report successful deletion of the CTG repeat expansion with CRISPR-cas9 and transcriptional downregulation of the toxic mRNA with dCas9. C. Huntington’s Disease (HD) is a CAG nucleotide repeat expansion disease. Mutation in the *HTT* gene leads to a systemic accumulation of an elongated HTT protein. The accumulation in the striatum of the brain gives the typical triad of symptoms: chorea, psychiatric disorders, and cognitive impairment. Studies show reversal of the neurotoxic effects after CRISPR-cas9-induced silencing of the *mHTT* in mice.

Many research groups report successful CRISPR-cas strategies to edit *DMD* pathogenic variants, *in vitro*, *in vivo*, and *ex vivo*. Most of them aimed to restore the reading frame of the *DMD* locus by deletion of the exon(s) where a pathogenic variant has been located (Fig 4). This would then restore the expression of a shorter form of dystrophin in muscle cells, converting DMD to BMD. The idea was derived from the development of exon-skipping therapies that use antisense oligonucleotides (AOs). AOs are synthesised DNA molecules that in the case of DMD are designed to interfere with splicing at mRNA level in order to restore a truncated form of dystrophin. The limited, though promising, success with pre-clinical development of AOs therapies paved the way for the first clinical trials that showed unsatisfactory results. [41] Even if the efficacy of AOs therapy improves, it would still not be an optimal approach to treat DMD, because it requires life-long administration of AOs, with potential toxic effects. [42–44] Genome editing could be a better approach in editing the *DMD* locus–it might require just a one-time delivery of CRISPR-cas systems and offer technical advantages by allowing excision of multiple exons with only one or two gRNAs. If successful, genome-editing could induce permanent exon skipping in patients’ cells.

Indeed, different authors report successful *DMD* editing and restoration of the reading frame, resulting in expression of dystrophin in human and mouse cells, treated *in vitro* and *in vivo*. The authors of numerous publications report NHEJ-induced skipping of: exon 2 [45], exons 3–7, exons 6–7, exons 7–11 [46], exons 18–30 [47], exons 21–23 [48], exon 23 [23, 49–55], exon 45 [56], exon 51[23, 39, 57, 58], exon 53 [58, 59], exons 48–50 [23], exons 52–53 [59], exons 51–53 [60, 61], exons 44–54 [58, 62], and exons 45–55 [44, 63]. These strategies cover the majority of the *DMD* mutations in the two hotspot regions.

Nonetheless, approaches differing from deletion-induced exon skipping have also been explored. Some research groups have managed to induce HDR and restore the full-length *DMD* gene in cells and mice, though with low efficiency. [50, 56, 59, 64, 65] Others have used catalytically-inactive, dead cas9 (dcas9) to epigenetically upregulate the expression of utrophin [47, 66] and laminin subunit α1 [67]. The authors argue that upregulation of these proteins strengthens the dystrophin-glycoprotein complex and its binding to the extracellular matrix, thus reducing the mechanical stress during force generation and improved functionality of the cells.

#### Efficacious editing in skeletal muscles

Studies report varying proportions of restored dystrophin in both skeletal and heart muscle cells. The *in vivo* studies also assessed the effect that NHEJ- and HDR-induced editing of the *DMD* gene and subsequent restoration of the dystrophin protein has on functionality of muscles in postnatal mice.

Nelson *et al*. (2016) managed to restore dystophin in CRISPR-cas9-treated muscle cells of *mdx* mice (model of DMD with cC3185T mutation in exon 23) up to ~8% of normal levels. Immunofluorescent staining showed that ~67% of cells expressed dystrophin. This was sufficient to improve muscle structure and function in tibialis anterior muscles (TAM) of *mdx* mice. [53] Tabebordbar *et al*. (2016) report similar findings, with restored dystrophin levels ranging from 3–18% in different groups of skeletal muscle cells and cardiomyocytes of *mdx* mice. [54] Xu *et al*. (2016), along with the previous two groups, were among the first ones who demonstrated successful dystrophin restoration in live postnatal *mdx* mice, reporting levels of up to 50% of those of WT. Furthermore, they confirmed that CRISPR-cas9-mediated dystrophin restoration improved the functionality of the dystrophin-glycoprotein complex by ameliorating localization of proteins, thus recuperating sarcolemmal integrity. Long *et al*. (2016) went on to address how the mode of systemic administration (injection) of CRISPR-cas9-containing AAV 9 viral genomes might also affect the efficacy of dystrophin rescue. They report that highest restoration of dystrophin was observed after intramuscular delivery (IM), with 25.5% ± 2.9% of TAM myofibres expressing the protein. Other modes were less efficient–intraperitoneal (IP) delivery of CRISPR-cas9 systems to *mdx* mice rescued dystrophin in 1.8 ± 1.2% of TAM myofibres and 3.2 ± 2.4% of cardiomyocytes (8 weeks after delivery), whereas retroorbital (RO) delivery rescued dystrophin in 4.6 ± 3.2% of TAM myofibres and 9.6 ± 3.9% of cardiomyocytes (12 weeks after delivery). Semiquantitative immunohistochemistry confirmed that dystrophin protein levels were highest in skeletal muscle cells in mice with IM delivery of CRISPR-cas–up to 53.2% of dystrophin levels in wild type (WT) mice. However, they report that RO delivery might be more efficient in rescuing dystrophin in the heart. [50]

Amoasi *et al*. (2017) report a higher efficiency, with more than 90% restoration of dystrophin in skeletal muscle cells, after injection of CRISPR-cas9 systems in the TAM in young mice lacking exon 50 - *ΔEx50*, a new DMD animal model they created for the purpose of this study. Western blotting confirmed that the levels of dystrophin in treated *ΔEx50*-mice were restored to approximately 80% of those of WT mice. After systemic AAV delivery of cas9 and one gRNA targeting exon 51 in young *ΔEx50* mice, they detected an extensive expression of dystrophin in the heart, TAM, triceps, and diaphragm muscles. Furthermore, the treated *ΔEx50* mice had a substantial improvement in histopathological morphology of muscle cells, muscle grip strength, and creatine kinase (CK) levels. [39] Bengtsson *et al*. (2017) reinstated dystrophin expression in the heart (34% of cardiomyocytes) and skeletal muscle cells, four weeks after AAV 6 systemic delivery of CRISPR-cas9 systems in young *mdx*^*4cv*^ mice. The treatment also improved muscle force generation. They demonstrated a dose-dependent increase in dystrophin levels in cardiac cells with both high (1 x 10^13^ v.g.) and low dose (1 x 10^12^ v.g.) viral loads, but reported that only the higher dose of viral delivery led to widespread dystrophin restoration, up to 70% (<10% in quadriceps and extensor digitorum longus muscles to >50% in soleus muscles). The treated *mdx*^*4cv*^ mice showed improvement in force generation, in comparison to controls. [59] Lee *at al*. (2017) managed to pack the cas9 protein, along with a gRNA and a donor DNA template, into a novel design of gold nanoparticles, which they named CRISPR-Gold. They demonstrated that after an intramuscular injection in *mdx* mice of 6 mg/kg CRISPR-Gold, simultaneous with cardiotoxin, led to 5.4% HDR-induced correction of the mutated *DMD* to WT sequence. This not only restored dystrophin in muscle cells, but also improved muscle strength and agility, with treated mice having “two-fold increase in hanging time in the four-limb hanging test” compared with controls. [64] They used cardiotoxin to induce tissue damage and subsequent proliferation of muscle stem cells. This might have contributed to the higher efficiency of HDR.

#### Editing heart muscle cells

The *in vivo* studies mentioned above have assessed and reported only functional improvement of skeletal muscles. Although some of them report restored dystrophin levels in heart muscle cells after systemic delivery of CRISPR-cas, it remained unclear whether dystrophin restoration in cardiomyocytes can also improve heart function. El-Refaey *et al*. (2017) were the first group to report that CRISPR-cas9-induced dystrophin restoration (up to 40%) in the heart muscle improves the cardiac structure and function. They used AAV rh74 to deliver CRISPR-cas9 systems to *mdx/Utr*^*+/−*^ mice (*mdx* model with utrophin haplodeficiency), using a retroorbiral mode of viral injection. Ten weeks after delivery they isolated the papillary muscles from *mdx/Utr*^*+/−*^ mice and demonstrated that CRISPR-cas9 treated mice had improvements in the histopathology and contractility, compared to controls. [49]

#### Epigenetic manipulation

Catalytically inactive dCas9 has been used for epigenetic upregulation of proteins that improve sarcolemmal stability, such as utrophin and laminin subunit α1, in postnatal *mdx* mice. These proteins are not expressed in the adult muscle tissues, but might prove efficacious in improving muscle strength. [66, 67]

#### The efficiency levels might be attributable to gRNA and cas protein

As expected, the differences in CRISPR-cas efficacy are likely attributable to the different approaches to editing the *DMD* locus as well as the models of disease. Overall, using a dual-cleavage approach, with two gRNA molecules–one upstream and one downstream of the sequence to be deleted–showed higher deletion efficacy and higher precision in deletion of the targeted sequence, compared to using just one gRNA. Most of the authors used the cas9 derived from *Streptococcus pyogenes* (SpCas9), whereas some of them used cas9 derived from *Staphylococcus aureus* (SaCas9). The two seem to show no differences in editing efficacy. [49, 59, 62] However, Zhang *et al*. (2017) demonstrated that their new cas protein–cpf1– can also successfully edit the *DMD* locus. The authors report successful deletion of exon 50 in human cardiomyocytes derived from induced pluripotent stem cells (iPSCs), as well as HDR- and NHEJ- germline editing of exon 23 in *mdx* mice. The study reports that cpf1 endonuclease seems as efficacious and safe as the cas9, expanding the editing repertoire through targeting T-rich PAMs and leaving sticky ends. The authors hypothesise that cuts with sticky ends might improve the NHEJ-mediated deletion.

#### Specificity

Most of the authors assessed the specificity of the CRISPR-cas systems by analysing the sequences of potential off-target loci, predicted using bioinformatic tools. Frequently used were the T7 endonuclease 1 assays (T7E1) and sequencing to assess the sequences of these potential off-target loci and report little or no unspecific changes in the latter, induced by treatment with either CRISPR-cas9 or CRISPR-cpf1. However, Li *et al*. (2015) did a whole-genome analysis on CRISPR-cas9-treated iPSCs and found that indels occurred slightly more often in CRISPR knock-in clones compared to controls. [56] Still, they conclude that no severe off-target mutagenesis occurred because the indels were mainly located in triple-repeat or GC-rich regions, consistent with findings of others [68, 69]. Long *et al*. (2014) discuss that the estimated frequency of off-targets might be lower for *in vivo* studies compared to *in vitro* studies. However, the safety question due to unwanted off-targets remains open, until genome-scale unbiased assessment of off-target activity becomes a methodological practice in outcome assessment of CRISPR-cas9 specificity. [70]

#### The delivery is predominantly viral

Studies on cells and *ex vivo* strategies used different viral and non-viral delivery of CRISPR-cas9 systems (Table 1). Muscle cells are notorious for their difficulty in transfection. [71] Most of the research groups report that electroporation was the only method that yielded good transfection efficiency in myoblasts (including nucleofection as a specific electroporation technique).

Viral delivery remains the preferred and most investigated method of delivery *in vivo* (Table 1). Some of the groups used AdV as a method of CRISPR-cas9 delivery for *in vivo* and *ex vivo* studies, due to its large packaging capacity. Although efficient, AdV induces a strong immune response and a large proportion of the human population is already immune to it. [72] Ehrke-Schulz *et al*. (2017) have addressed this issue and were the first group to modify high-capacity adenoviral vectors (HC-AdV) for easier manipulation and delivery of CRISPR-cas9 systems. [57] They demonstrated the editing efficacy of CRISPR-cas9 delivered by HC-AdV to primary human skeletal myoblasts. Furthermore, they claim that HC-AdV might be more advantageous over other methods of delivery, referring to the previous work of other research groups. [73–75] Namely, HC-AdV have a large packaging capacity (up to 35 kb), they do not express any viral genes that might trigger an immune response, and they have an improved genotoxicity profile compared to other viral delivery systems because they remain outside of the host chromosomes (lentiviruses incorporate into the host chromosomes, whereas AAV partially do so). [57, 76] Although promising, the efficacy of HC-Adv delivery is yet to be confirmed *in vivo*.

Still, the AAV was most frequently used for treating mouse DMD models, due to its ascertained and reported safety. The AAV induces low, negligible immune response and is not pathogenic for humans even in the WT form. [77] Most research groups chose AAV serotype 9, in order to improve delivery to the heart and skeletal muscles, but some of them used AAV serotypes 6, 8, and rh74. The main drawback of AAV is its limited packaging capacity (~ 4.7 kb). If using SpCas9, AAV have enough space for inserting one gRNA. If two or more gRNAs or other genes require delivery, then usually dual AAV vector delivery was required (one AAV carrying the SpCas9 and one for the gRNAs or other cassettes). Some of the researchers circumvented this limitation by using the shorter SaCas9, saving around 1 kb of space for other elements. In order to enhance expression of CRISPR-cas9 systems specifically to muscles and the heart, Amoasi *et al*. (2017) [39] and Bengtsson *et al*. (2017) [59] used a CK8 muscle-specific cassette in the AAV. This might explain why they achieved higher efficiency in dystrophin rescue compared to others.

#### Non-viral delivery

CRISPR-Gold appears as a new non-viral method of delivery. Lee *et al*. (2017) demonstrated that gold nanoparticles are efficacious in delivering in its protein form and the gRNA in RNA form. This might allow better control over dosage administration. They assessed the immunogenicity of CRISPR-Gold, reporting no change in inflammatory cytokine levels in the plasma of mice, 24 hours and two weeks after treatment. However, the treated muscle tissues had higher numbers of CD45+ CD11b+ cells compared to controls. The authors argue that although these cells are reported in muscle tissues undergoing inflammation and regeneration [78], their appearance is likely attributable to the macrophage-mediated clearance of the nanoparticles and microparticles. Eventually, they demonstrated that plasma cytokine levels do not increase even if CRISPR-Gold is injected in mice twice, with a dosing time interval of three days.

#### Translating the findings from the bench to the bedside

Although the *ex vivo* strategies do show potential, ideally, they would not be the most pragmatic approach for DMD treatment in humans. They would involve many steps—isolating iPSCs from patients, propagating them *in vitro*, editing them with CRISPR-cas9 and then transplanting them back to the patients–each of them carrying their own technical and practical hurdles. Furthermore, it would increase the costs of the treatment. Based on current evidence, systemic *in vivo* delivery of CRISPR-cas9 systems may be the most promising strategy with regard to efficiency. However, controlling and manipulating the CRISPR-cas9 systems *ex vivo* is easier. More importantly, examining the potential toxic effects prior to transplanting the treated cells inside the patient makes the *ex vivo* strategies a safer option to start the development with.

Although most of the preclinical studies focus on permanent exon skipping by CRISPR-induced deletion, this would not be a causative treatment for DMD. It would likely improve the symptomatology in patients, transitioning them to a milder BMD-like phenotype, but would not cure the disease. Optimal strategy would be HDR-induced restoration of full-length dystrophin. Unfortunately, at the moment CRISPR-cas9-induced HDR shows low efficiency. Until the HDR-efficiency improves, perhaps CRISPR-cas9 deletion of exons and their subsequent skipping would have most merit for exploration in clinical trials. Still, we need better, standardised, and reliable methodological approaches in CRISPR-cas gene editing before we approach design and execution of clinical trials. We would argue that CRISPR-cas should not reach the phase of clinical testing until more studies explore the specificity and mutagenesis with off-target events, using whole-genome analysis. In addition, we recommend all *in vivo* studies to adhere to the ARRIVE (Animals in Research: Reporting *In Vivo* Experiments) guidelines. [79]

Delivery still remains a major bottleneck. Based on current evidence, AAV-mediated delivery appears as the most evidence-based approach for clinical trials. Genome editing-based treatments delivered at young age of patients would prove more efficient, probably due to positive selection of gene-edited cells in muscle tissues. This would also require improvements in diagnostics, since most of the DMD patients are diagnosed at the age of 3–5 years old. It is likely to obtain higher editing efficiency in both the heart and the skeletal muscles using dual vectors of AAV serotype 9, with the CK8 muscle specific cassette. The viral vectors carrying the CRISPR-cas components might show better systemic delivery with intramuscular administration. Still, previous reports have shown immune response towards cas9, especially after intramuscular delivery. In addition, the question whether the viral load dosages are translatable to humans raises another concern. [80]

Beside the disadvantages of AdV delivery that we have discussed above, they are additionally questionable for approval in clinical trials due to an unfortunate event in the 90s, when an 18-year-old patient with hereditary enzyme deficiency died 4 days after gene therapy with AdV vectors. [81] As discussed before, HC-AdV delivery might be an advantageous improvement that should be further explored *in vivo*.

Although non-viral delivery systems are poorly explored, the study of Lee *et al*. (2017) brings excitement in the research community due to several reasons. Gold nanoparticles seem promising in *DMD* treatment, because they allow the delivery of CRISPR-cas components in different forms *via* endocytosis, seem safe with regard to eliciting immune response, provide better control over the dosage, and allow multiple injections of the treatment. It is plausible that multiple injections of CRISPR-cas9 systems might lead to higher efficiency in dystrophin rescue and improvement of muscle strength, because it would edit more pluripotent stem cells present in the muscle tissues at different time points. Gold nanoparticles might be more efficient in gene editing using an NHEJ strategy. More development and exploration of non-viral delivery systems is required in the years to follow.

### Myotonic dystrophy (DM)

Myotonic dystrophies are the most prevalent neuromuscular disease in adults. They usually present with myotonia, muscle weakness and wasting, conduction defects in the heart, restrictive pattern of respiratory insufficiency, cataracts, and endocrine disorders. Two forms are described: type 1 (DM1) and type 2 (DM2). [82]

DM1 is more prevalent, with an estimated prevalence of ~ 1 in a population of 8,000. [83] The disease leads to premature death, with median age at death of 56 years, observed in cohorts in Canada and the Netherlands. [84, 85] Probability of survival after the age of 65 years is low. Most patients die from respiratory failure or pneumonia, cardiovascular disease, sudden death, and neoplasms. [86] Although underdiagnosed, DM2 seems less prevalent than DM1. Overall, it is milder, with later onset and longer life expectancy. [82]

#### Pathogenesis

DM1 is caused by a CTG trinucleotide repeat expansion in the dystrophia myotonica protein kinase (*DMPK*) gene, located on chromosome 19q13.3, whereas DM2 is caused by a CCTG tetranucleotide repeat expansion in intron 1 of the nucleic acid-binding protein gene (*CNBP*), also known as zinc finger 9 gene (ZNF9), located on chromosome 3q21. [87, 88] The two DM forms have different location of the nucleotide repeat expansions, but share the same RNA pathophysiological mechanism. The mRNA transcribed from these expanded gene loci produces a pernicious effect, referred to as “RNA gain of toxic function”. The expanded mRNA (mRNA^exp^), folded in hairpin-like structures, accumulates within the cell nucleus, forming accumulation of RNA and proteins termed ribonuclear foci. These structures sequester muscleblind-like family of proteins (MBNL) causing their loss of function. [89, 90] In addition, mRNA^exp^ causes upregulation of the CUBG and Elav-like family of proteins (CELF) leading to their gain of function. Both protein families belong to the group of RNA-binding proteins (RBPs), which regulate alternative splicing of pre-mRNA molecules. [91, 92] Their imbalance leads to splicing deregulation of many genes, including muscle-specific chloride channel (*CLCN1*), insulin receptor (*IR*), bridging integrator 1 (*BIN1*), pyruvate kinase M (*PKM*), troponin T (*TNNT2*), and many others. At the end of the chain of events, the mis-splicing of genes leads to translation of dysfunctional proteins that defines the clinical presentation of the disease. For example, deregulated splicing of the *CLCN1* causes reduced permeability for chloride ions in the sarcolemma, contributing to myotonia—delayed relaxation after voluntary muscle contraction, and a core symptom of DM. The insulin resistance is attributable to the mis-splicing of *IR*. The *BIN1* protein contributes to T-tubule network organisation and facilitates contraction in skeletal muscles; its mis-splicing seems to lead to muscle weakness. [93]

#### CRISPR-cas editing strategies

Few groups report successful gene-editing in DM. Ideally, one would aim for excision of the expanded nucleotide repeats in the *DMPK* or *CNBP* gene, followed by HDR-mediated repair and replacement with a sequence of normal size (see Fig 4). Long repeats are difficult to delete and HDR has low efficacy. However, it seems that complete removal of the *DMPK* gene does not affect the physiology of the cells. Accordingly, van Agtmaal *et al*. (2017) report successful excision of the *DMPK* locus in a cellular model of DM1, using dual cleavage approach. [94] Furthermore, they report that *DMPK* deletion led to complete clearance of the ribonuclear foci in the nuclei of the treated myoblasts, due to the absence of mRNA^exp^ to sequester the MBNL proteins. Analysis of the predicted off-target loci showed no changes in their sequences in the treated cells. Batra *et al*. (2017) tried a different approach which could be implemented in other nucleotide repeat expansion diseases as well. They demonstrated that modified dCas9 can be used to visualise and target mRNA^exp^ in modified COS-M6 cells. Previously they transfected the COS-M6 cells with plasmids containing (CTG)^105^, (CCTG)^300^, (GGGGCC)^120^ nucleotide repeat expansions, to represent molecular phenotypes of DM1, DM2, and HD, respectively. They used this modified dCas9 to target and remove mRNA^exp^ in DM1-patient-derived myoblasts and succeeded in removing more than 90% of the ribonuclear foci in the treated cells. Furthermore, they showed that the removal of the foci restored the localisation of the MBNL proteins and regulated splicing in myoblasts. [95]

Pinto *et al*. (2017) went on to explore the strategy on regulating mRNA^exp^ transcription *ex vivo* and *in vivo*. Five weeks after AAV-dcas9-gRNA administration by temporal vein injection in *HSA*^*LR*^ mice (a model of DM1), they performed electromyography in TAM and gastrocnemius muscles to assess myotonia. AAV-dcas9-gRNA-treated *HSA*^*LR*^ mice showed reduction in myotonia, compared to controls. Most of the treated mice showed positive myotonia signals of 33–60% of needle insertions, whereas the control *HSA*^*LR*^ mice usually had myotonia signals of more than 80% of needle insertions. In theory, improvement in myotonia should have been accompanied by corrected splicing of the *CLCN1* gene. In spite of improvement of the phenotype of treated mice, the authors did not achieve reduction of mis-splicing events of *CLCN1*, arguing that a rescue of some regions in the muscles, but not others, might have contributed to the discrepancy in the results. [96]

#### Translating the findings from the bench to the bedside

Out of the two strategies, transcription regulation of mRNA^exp^ might be easier to achieve than excision of larger repeats in *DMPK* or *CNBP* genes. This strategy would require repeated life-long administration of CRISPR-dcas9 systems, with effects still not accounted for. To this date, we do not know how this approach might affect the transcription of other genes. CRISPR-cas9-induced permanent removal of the nucleotide repeat expansions would be an optimal approach. Though, van Agtmaal *et al*. (2017) demonstrated that full excision of the *DMPK* locus does not affect the function of the cells. Therefore, the aim should be an HDR-induced replacement of the allele with a normal-sized repeat length. More *in vivo* studies are required in order to demonstrate the efficacy and safety of either approach. Notwithstanding the scarcity of the evidence, CRISPR-cas9 seems a promising therapeutic approach in DM.

### Limb-girdle muscular dystrophy (LGMD)

LGMD is an umbrella term for a group of Mendelian disorders that share a similar clinical presentation, i.e.—progressive proximal muscle weakness. The presentation varies from mild disease with normal lifespan to severe neuromuscular disease that also affects the heart and the respiratory muscles, causing premature death. Although the LGMD phenotypes are similar in presentation, the causing pathogenic variants differ in their type and chromosomal location. Due to improved diagnostics with next-generation sequencing, new genes causing LGMDs are discovered every year, with at least 31 disease-loci identified until today. LGMDs are classified in two groups: autosomal dominant LGMD1 and autosomal recessive LGMD2. LGMD2 mutations account for most of the disease phenotype, with an estimated cumulative prevalence of 1 in 15,000 individuals. [97] An *in vitro* proof of concept for gene-editing of LGMD2B and LGMD2D has been reported.

#### Pathogenesis

LGMD2B is caused by missense or null mutations in the dysferlin gene (*DYSF*) and subsequent loss of function of dysferlin, a sarcolemmal transmembrane protein responsible for skeletal muscle membrane repair. On the other hand, mutations in the α-sarcoglycan gene (*SGCA*) cause function loss of the α-sarcoglycan protein in the sarcolemma. Dysfunctional or absent α-sarcoglycan brings instability in the costameres, causing the more severe disease phenotype that is LGMD2D. [98]

#### CRISPR-cas editing strategies

In a proof of concept, Turan *et al*. (2016) combined CRISPR-cas9 with single-stranded oligonucleotides (ssODNs) to induce HDR and edit point mutations that cause LGMD2B and LGMD2D. In patient-derived iPSCs, they corrected the c.5713C>T mutation in the *DYSF* gene and the c.229C>T mutation in the *SGCA* gene. Opposite to their expectations and previous reports, they obtained higher editing efficiency using one gRNA (6.6% for *DYSF* and 3.5% for *SGCA*) compared to dual gRNA approach (1.39% for *SGCA*). They report little or no off-target editing in the predicted off-target loci, as observed with the T7E1 assay. [99]

### Facioscapulohumeral muscular dystrophy (FSHD)

FSHD is one of the most prevalent dystrophies. It was estimated that FSHD affects 1 in 20,000 individuals. However, a recent study in the Dutch population suggests that it might be twice as frequent in the population. It is likely underdiagnosed, due to its varying presentation and complex genetic pathophysiology. Patients with FSHD have progressive weakness and wasting of muscles of the face, shoulder and upper arm. The muscular atrophy is usually asymmetric, and the disease varies from very mild phenotypes to severe forms with early onset. [100]

#### Pathogenesis

The genetic background of FSHD is quite peculiar. The autosomal-dominant FSHD (responsible for ~ 95% of the phenotypes) is associated with contractions of D4Z4 macrosatellite repeats at chromosome 4q35. A length of 11–100 D4Z4 repeats on both chromosomes 4 is present in healthy individuals, whereas FSHD patients have contraction to 1–10 D4Z4 repeats on only one chromosome (4qA, where the contraction is in *cis* position with the disease-permissive haplotype), but not on the other one (4qB, the contraction does not lead to disease). Chromosome 10 contains an identical D4Z4 repeat on its subtelomere (10q26), but its contraction also does not lead to FSHD. The reason behind it lies in the *DUX4* gene, a transcription regulator. All three D4Z4 repeats contain a copy of the *DUX4* gene, however only the repeat that is most distal of the three (4qA) allows production of a full-length DUX4 protein. The expression of this aberrant DUX4 protein in muscle cells targets the expression of germline genes, immune mediators, and retroelements, alters the metabolism of RNA and proteins, and causes apoptosis of muscle cells, which eventually leads to muscular atrophy. [101]

#### CRISPR-cas editing strategies

Himeda *et al*. (2016) developed a therapeutic strategy based on dcas9 fused to transcriptional effectors in order to epigenetically regulate *DUX4* gene expression. They managed to induce heterochromatin formation and correspondingly decreased the expression of the full-length *DUX4* gene mRNA. This reversed the pathophysiological effects of the disease in primary FSHD myoblasts, suggesting that this approach might be a potential CRISPR-cas9-based treatment of the disease. They further argued that this approach might be safer because it does not induce cuts within the cellular DNA. [102]

### Congenital muscular dystrophy type 1A (MDC1A)

#### Pathogenesis

MDC1A is caused by mutations in the *LAMA2* gene, which lead to α2 chain (Lama2) deficiency within the laminin-211 protein complex, an important component of the extracellular matrix. The interaction of laminin-211 complex with proteins in the sarcolemma (integrins α_7_β_1_ and dystroglycans) is lost in MDC1A, leading to damage of the basement membrane of muscle and Schwann cells, muscle tissue degeneration and fibrosis. The levels of Lama2 deficiency are associated with disease severity. MDC1A is diagnosed in 1–9 individuals in a population of one million. It usually presents in neonatal age, with severe muscular hypotonia, generalised muscle weakness, scoliosis, increased creatine kinase, and demyelinating neuropathy. Most patients die before the age of 10 years old. [103]

#### CRISPR-cas editing strategies

A demonstration of the potential of gene-editing in MDC1A treatment was demonstrated by Kemaladewi *et al*. (2017), who delivered CRISPR-cas9 systems *via* AAV 9 vectors to delete a point mutation in intron 2 of the *LAMA2* gene in *dy*^*2J*^*/dy*^*2J*^ mice (a model of MDC1A). They report that selective *in vivo* delivery of CRISPR-cas9 systems to the muscles was inefficient in improving muscle strength in *dy*^*2J*^*/dy*^*2J*^ mice, likely due to remaining nerve demyelination caused by degenerated, unedited Schwann cells. However, systemic delivery at day 2 of life of AAV9-cas9-gRNAs, targeting intron 2 of the LAMA2 gene, through injection in the temporal vein of *dy*^*2J*^*/dy*^*2J*^ mice restored full-length Lama2 proteins in muscle and nerve cells, 30 weeks after treatment. They detected improvement in the histopathology of the diaphragm, TAM, and gastrocnemius muscles, as well as myelination of the sciatic nerve. As a result, the treated *dy*^*2J*^*/dy*^*2J*^ mice had improved muscle activity, with hind-limb and dorsi flexor muscles’ strength reaching almost full strength of WT mice. Furthermore, the treated mice had improvement in the degree of paralysis and managed to walk a distance twice as far as controls, and close to that of WT mice. They report no off-target events, as detected with the T7E1 assays. This demonstrates that CRISPR-cas9 can be used to restore Lama2 levels in both muscles and nerves, which is required to reverse the phenotype of MDC1A. [104]

### Huntington’s disease (HD)

HD has been in the focus of neurologists and researchers since the late 19^th^ century. Still, no cure has been developed so far, despite the numerous efforts. A new promising approach might be AOs-based treatments, which are now undergoing clinical trials. [105] It affects around 1 in 7,300 individuals, as estimated in Western countries. On average, it appears in midlife, at the age of 45 years old, usually with motor symptoms onset. Half of the patients die within 18 years after the primary symptoms have occurred. [106]

#### Pathogenesis

An autosomal-dominant disease, HD is caused by CAG nucleotide repeat expansions in the huntingtin (*HTT*) gene, located on chromosome 4q16.3 (see Fig 4). [107] This mutated *HTT* gene (*mHTT*) encodes a toxic, elongated mutated huntingtin protein (mHTT) that accumulates in the cells, leading to cellular death. Although the toxicity is systemic, accumulation in the brain—particularly in the striatum—causes the typical triad of symptoms: motor dysfunction (typical symptom is chorea–quick, irregular, uncontrolled movements of the hands or feet), cognitive impairment (attention deficit and inability to recognise emotions), and neuropsychiatric features (frequently apathy). The severity of the disease is mainly dictated by the length of the CAG repeats–the longer the repeat expansion, the more severe the disease is. [108]

#### CRISPR-cas editing strategies

HD has reached the *in vivo* phase of exploratory preclinical studies, with two groups reporting successful *mHTT* gene-editing in mouse models of the disease.

Shin *et al*. (2016) were the first ones who used CRISPR-cas for *in vitro* patient-personalised, allele-specific editing of the *mHTT* gene. After observing individual variation in the *mHTT* gene of patient-derived primary fibroblasts, they have detected single nucleotide polymorphisms in PAM specific for the *mHTT* gene. This allowed them to use CRISPR-cas9 to target pairs of PAM sequences in the mutant haplotype in order to inactivate it, while preserving the wild type *HTT* allele. Furthermore, they managed to delete 44kb of DNA encompassing the *mHTT* allele in iPSC lines. [109] Kolli *et al*. (2017) reduced transcription of mHTT gene by 80% in mesenchymal stem cells extracted from *YAC128* mice by CRISPR-cas-targeting of the promoter region upstream from the CAG nucleotide expansion portion of exon 1. [110] Xu *et al*. (2017) also demonstrated a successful editing of the *mHTT* gene in iPSCs followed by reversal of phenotypic abnormalities in the cells. Furthermore, they managed to differentiate them to excitable, synaptically active forebrain neurons. [111]

Transferred to *in vivo* models, Monteys *et al*. (2017) were the first group to demonstrate a proof of concept for CRISPR-cas9 treatment in *BacHD* mice (a model of HD, which carries the human *mHTT* gene). Like Kolli *et al*. (2017), they also aimed for silencing the *mHTT* gene through deletion of exon 1. For this purpose, they injected AAV vectors carrying CRISPR-cas9 components in the right brain hemisphere of *BacHD* mice. Three weeks later, the harvested DNA from the two brain hemispheres showed deletion of exon 1 in the *mHTT* gene in the striatum from the right hemisphere, but not in the left striatum. This was followed by 40% reduction in *mHTT* expression in the right hemisphere. The authors report no changes in the predicted off-targets’ sequences as assessed by Sanger sequencing. [112]

Yang *et al*. (2017) report successful non-allele specific deletion of the *mHTT* in the striatum tissue. To experimentally simulate the treatment *in vivo*, they used AAV vectors to deliver CRISPR-cas9 systems to heterozygous 9-month-old *HD140Q-KI* mice (a model of HD), which lead to *mHTT* deletion in somatic cells, and reversal of the neurotoxic effects of the HTT aggregates and the behavioural patterns of the disease model. The treated mice had improvement in rotarod, balance beam and grip strength tests, compared to controls. Whole-genome sequencing of DNA extracted from the striatum of treated mice showed that the off-target events occurred mainly in proximity of the sequences targeted with the gRNAs. They did not detect mutations in the potential off-target loci, which was additionally verified with T7E1 assays. [113]

#### Translating the findings from the bench to the bedside

It is exciting to see that *HD140Q-KI* mice treated with CRISPR-cas9 in their adulthood have reversal of the *mHTT* neurotoxicity. This suggests that *mHTT* gene-editing after the onset of symptoms still allows neurons to clear up the huntingtin protein aggregates. In addition, the improvement in the phenotype of the treated *HD140Q-KI* mice shows promise for future development of CRISPR-cas9 therapeutic strategies in HD. Yet, the specificity of the CRISPR-cas9 gene editing still remains an issue. Although the sequence analyses show that no editing occurs in the predicted off-target loci, the evidence that off-target activity occurs in proximity of the targeted sequences raises a concern. Once again, we would emphasize the importance of ensuring the safety and precision of CRISPR-cas9 systems in gene-editing, before considering clinical trials.

### Fragile X syndrome

Fragile X syndrome (FXS) is the most frequent hereditary cause of intellectual disability and one of the most frequent monogenic causes of autism. FXS is inherited in an X-linked dominant manner. With penetrance of 80%, FXS affects 1 in 4,000 men. FXS has a lower penetrance of 30–50% in women, as well as lower prevalence of 1 in 7,000 women. Men are usually more severely affected. [114]

#### Pathogenesis

The pathogenic variant responsible for 98% of the disease phenotype is located in the fragile X mental retardation protein (*FMR1*) gene, located at Xq27.3. It is a CGG nucleotide repeat expansion, leading to methylation of the repeats as well as the adjacent CpG island. The silencing of the *FMR1* gene leads to absence of both transcription and subsequent translation of the FMR1 protein. The FMR1 protein is normally expressed ubiquitously in the early postnatal period and throughout life, mainly in the brain and spermatogonia. Its role in regulating expression of genes involved in neuronal synapses is well established. Thus, the absence of FMR1 protein, through mechanisms not yet known, typically presents with intellectual disability, combined with dysmorphia (long face, hyperextensible joints, and prominent ears) and post-pubertal macroorchidism. The patients may have additional clinical presentation with: psychiatric disorders, fibromyalgia, sleep disturbances, pain, and other neurological syndromes. [115]

#### CRISPR-cas editing strategies

Two groups report successful excision of the CGG nucleotide repeat expansions in the *FMR1* gene. Both Park *et al*. (2015) and Xie *et al*. (2016) used CRISPR-cas9 to target the pathogenic variant and reverse the expression of FMR1 protein in cellular models of FXS. [116, 117]

#### Translating the findings from the bench to the bedside

Although interesting as proofs of concept, it is unclear how CRISPR-cas would be implemented as a therapeutic approach in FXS *in vivo*. Substantial evidence exists for plasticity in the developed brain; but we do not know if effects of the FMR1 protein induced in early development, before birth and during the first two years of life, can be compensated with reactivation of the *FMR1* gene later on. It seems unlikely that reactivation of the *FMR1* gene with CRISPR-cas could reverse the phenotype of those patients with typical and severe presentation of the disease.

### Friedreich’s ataxia (FRDA)

FRDA presents early in life (adolescent age) with ataxia and areflexia. Dysarthria, hearing difficulties, and nystagmus may also appear. Non-neurological affection includes cardiomyopathy, diabetes, and typically scoliosis. On average, it appears at the age of 16 years old, and the patients are wheelchair bound 11–15 years after the onset. [118] Half of the patients die within 36 years after the onset of disease. Onset before or at 20 years of age and cardiomyopathy are associated with shorter survival rates. [119]

#### Pathogenesis

FRDA is a rare autosomal recessive disease, caused by GAA repeats expansions in the frataxin gene (*FXN*), located on chromosome 9q21.11. A homozygous mutation in the *FXN* gene leads to epigenetic deregulation and reduced expression of the frataxin protein to levels 5–35% of those of healthy individuals. Frataxin is involved in the function of mitochondria. Dysfunction in the respiratory chain of the cells causes cellular degeneration. Degenerative changes in the dorsal root ganglia of the spinal cord, peripheral nerves, the cerebellum, and sometimes the vestibulum, lead to the typical presentation of the disease. [120]

#### CRISPR-cas editing strategies

Ouellet *et al*. (2017) have successfully managed to delete the repeat expansions within the *FXN* gene in fibroblasts derived from *YG8R* and *YG8sR* mice (models of FRDA). However, contrary to their expectations, in fibroblasts derived from *YG8R* mice the deletion of the repeats led to reduced expression of frataxin. The authors report that this is likely attributable to the presence of two *FXN* transgenes in tandem in the genome of *YG8R* mice, leading to a deletion of not only the repeats, but of one entire copy. This eventually left only one *FXN* copy that was functional, explaining the fall in the frataxin levels. On the other hand, the deletion of GAA repeats in *YG8sR* mice increased the levels of frataxin in some clones of treated cells compared to corresponding controls. This further supports the explanation for the reduction in frataxin levels in treated *YG8R*-derived fibroblasts, because the *YG8sR* differs from the previous in a way that it has lost one of the *FXN* transgenes during reproduction. [121]

#### Translating the findings from the bench to the bedside

Reduction in frataxin levels with CRISPR-cas has been demonstrated in FRDA models of the disease. However, whether this can lead to reversal or improvement in the disease symptoms is yet to be investigated. Restoration of frataxin levels might not be of use if there is already a substantial degeneration of neural structures. Early delivery of CRISPR-cas systems that would successfully restore frataxin levels might perhaps prevent ongoing degeneration or stop the process is some neurons. Still, there is no evidence to suggest this claim.

### Spinocerebellar ataxia type 2 (SCA2)

SCA2 is caused by expansion of CAG repeats in the *ATXN2* gene. Although Marthaler *et al*. (2016) report successful gene-editing of three cellular models of SCA2 with the aim to create perfect controls for future studies, we included the studies in this review as evidence that this nucleotide repeat expansion disease can be edited with CRISPR-cas9 as well. They used CRISPR-cas9 to correct three clones of iPSCs models of SCA2 and created controls with a normal-sized *ATXN2* allele. However, they didn’t assess how this affected the expression of ATXN2 or any other subsequent physiological changes. [122–124] Except for the notion that CAG repeats expansions in the *ATXN2* gene are editable, we could not infer other information from these reports.

### Brief remarks on ethics: The individual in research and society

One could argue that discussing the ethical implications of a treatment that is not close to reaching the clinical setting might be like harvesting fruit that has barely flowered. However, the development itself of CRISPR-cas9 as a gene-editing tool with a potential to treat monogenic diseases already raises several ethical questions that need to be addressed.

#### Benefit-risk ratios

A common thread of thought appears in every development of a new treatment—whether the beneficial effects of the treatment overweigh the risks. [125] Although many authors report no major concerns over the off-target events appearing in human-cellular and animal models of neurogenetic diseases, the uncertainty of the findings due to the use of biased methods in assessment of the specificity of CRISPR-cas systems still leaves this question open. The higher frequency of indels in regions surrounding the targeted sequence gives additional concern for its safety. Furthermore, the variable efficiency and uncertainty in controlling the systems *in vivo* makes CRISPR-cas9 unreliable for moving towards clinical trials at the moment. Current evidence gives an unclear benefit to risk ratio, questioning the effect size of both. In the case of CRISPR-cas genome-editing, it seems like two benefit-risk ratios have to be considered: that of CRISPR-cas systems and that of the delivery systems. Failure in proper estimation of benefit-risk ratios for either of the systems would likely discourage both the research community and the public to implement CRISPR-cas in practice. [126]

#### Hope

An ethical dilemma on health communication and informed consent appears. If the scientific community cannot agree upon the strength of evidence for the efficacy and safety of a CRISPR-cas treatment, how can we expect that patients, who lack medical and research training, would contemplate all the pros and cons for receiving a new treatment in experimental phase? Many of the patients who suffer a debilitating disease with death in proximity are likely to be in a particular state of mind, willing to try any treatment that might have an effect. The hope that a new treatment might emerge could make them disregard the potential harms, questioning how informed the given consent is.

#### Germline editing

Plausible long-term effects of a CRISPR-cas treatment add another complexity in regard to informed consent. Potential unwanted or unwarranted changes in the genome would be additionally passed on to children who are not in a position to give an informed consent. If real, these long-term changes would make any genome-editing that would affect the offspring unacceptable. In order to assess long-term changes, study designs should include whole genome sequencing of the offspring of animal models treated with CRISPR-cas9, to clarify whether such concern is justifiable. Dr. Jeniffer Doudna, one of the pioneers of CRISPR-cas9, has asked for moratorium on human embryonic stem cells genome editing on several occasions. This was followed by numerous debates on germline editing and “designer babies” (genetically engineered children) emerged in the public, and the research and ethics committees. Eventually, the guidelines on ethical CRISPR-cas9 research were incorporated in the laws of most European countries, the United States, and Canada, banning human germline genome editing. Some of the leading scientists call upon the formation of a global surveillance system on gene-editing, considering the need for a universal moral ground on the matter. [127]

#### Rare diseases and the society

Most of the NMGDs have a low prevalence in the overall population. According to the definition of a rare disease as such of “a prevalence of not more than five affected persons per ten thousand”, all the diseases that we have discussed in this review are rare diseases. The current models of health economics in most of the countries in the world are governed by the principle that the needs of the many outweigh the needs of the few or the one. This brings a concern whether the costs of CRISPR-cas could prove cost-effective in practice, according to current economic standards. The scientific community should push forward new standards and develop new business models in order to maintain a potential CRISPR-cas treatment. This would require numerous efforts on many levels and multidisciplinary collaboration. Mulvihill *et al*. (2017) on behalf of the Committee of Ethics, Law, and Society (CELS) of the International Human Genome Organisation (HUGO) call upon solidarity and collaboration between research groups, in order to improve the speed and quality of research. [128]

#### Hypes and controversies

The discovery of CRISPR-cas split the public opinion. Misrepresentation by some researchers and mass media made some people believe that we will cure most of the genetic diseases in not so distant future. Some went on claiming that we are approaching a new, disease-free phase of human development. [129] Optimism in exaggeration seems harmless at first view, but it does affect the life of the individual, particularly of the patient who suffers from an incurable disease. In some patients, these hypes bring an exaggerated hope that makes the disappointment larger due to lack of applicability during their lifetime.

On the other side, some members of society have raised some unjustifiable fears. What seems as fearmongering has agitated the public that a new surge of eugenics is on its way and that genome manipulation would give birth to “designer babies”. [130] Benston (2017) gives an interesting comparison of this movement to that of the antivaccination lobby, with regard to its impact on society. [131] Vaccines brought a major change in the scientific community and the societies worldwide, thus becoming a political instrument used by some groups. CRISPR-cas has been widely applied in the scientific community. If it becomes applicable in practice for genome editing in humans, it might become a political instrument that should be carefully addressed. Benston (2017) believes that learning from the history of controversies surrounding vaccines, we might be better prepared for what might come in the future.

## Conclusions

CRISPR-cas is without doubt one of the most ingenious inventions of the 21^st^ century. It has already changed the way we do science and it is exciting to think of all the possibilities it could bring in the future. Much has been done in the first five years of CRISPR-cas development. Many improvements and modifications had made it possible for research groups all over the world to use CRISPR-cas systems to edit the genes of some of the most frequent NMGDs, both *in vitro* and *in vivo*. Their accomplishments are truly remarkable and stimulating in opening a new horizon of hope for the patients. Of all NMGDs addressed here, DMD is likely the first one to reach the clinic at some unclear point in the future, if all hurdles are gotten over.

That being said, at the current level of development, there is not enough evidence for CRISPR-cas transitioning to clinical trials for treatment of NMGDs in the near future. There are still many issues to consider before attempting the use of CRISPR-cas9 in patients. Overall, we would have to overcome three major obstacles: efficacy, safety, and delivery. The efficacy of CRISPR-cas is variable, while the control over its components, the dosage, metabolism and long-term effects *in vivo* unclear. Although off-target events seem to appear on rare occasions, this still remains an open question—most of the studies did not assess the whole genome for mutagenesis. Furthermore, some of the authors who did whole-genome analysis report a slight increase in indels in areas adjacent to the targeting sequences. Finally, the delivery remains an important difficulty to explore and solve. Viral vectors seem to be traditionally favoured due to their efficiency and level of investigation, whereas non-viral delivery systems seem to have just begun their journey to discovery.

The development of a CRISPR-cas treatment already raises some complex ethical concerns that need to be addressed and considered when designing both preclinical and clinical studies. Major efforts by numerous stakeholders within the society are needed for the optimal benefit of the individuals who suffer from these rare, monogenic neurological diseases.

## Supporting information

S1 Checklist Preferred Reporting Items for Systematic Reviews and Meta-Analyses (PRISMA). (PDF)

S1 Table Search keywords used for the systematic review. (DOCX)
