# Supplementary material for: CRISPR-cas gene-editing as plausible treatment of neuromuscular and nucleotide-repeat-expansion diseases: A systematic review
Source: PLoS One. 2019 Feb 22;14(2):e0212198. doi: 10.1371/journal.pone.0212198 (PMC6386526; doi:10.1371/journal.pone.0212198)
Supplement: S1 Table — (DOCX) [file pone.0212198.s002.docx]

**S1 Table.** **Search keywords used for the systematic review**

| Database | Search | Keyword variations 1 | Boolean operator | Keyword variations 2 |
| --- | --- | --- | --- | --- |
| MEDLINE | 1 | gene editing OR "gene editing" OR gene editing [Mesh] | AND | myotonic dystrophy OR "myotonic dystrophy" OR myotonic dystrophy [Mesh] |
|  | 2 |  |  | dystrophy OR muscular dystrophy [Mesh] OR "neuromuscular disease"  OR "neuromuscular disorder" |
|  | 3 |  |  | trinucleotide repeat expansion OR "trinucleotide repeat expansion"  OR trinucleotide repeat expansion [Mesh] |
|  | 4 |  |  | Huntington's disease OR "Huntington's disease" OR Huntington disease [Mesh] |
|  | 5 |  |  | spinocerebellar ataxia* OR "spinocerebellar ataxia" OR spinocerebellar ataxias [Mesh] |
|  | 6 |  |  | Friedreich* ataxia* OR "Friedreich ataxia" OR Friedreich ataxia [Mesh] |
|  | 7 |  |  | fragile X syndrome OR "fragile X syndrome" OR fragile X syndrome [Mesh] |
|  | 8 | CRISPR-Cas Systems OR CRISPR OR Cas OR Clustered Regularly Interspaced Short Palindromic Repeats OR CRISPR-Associated Proteins OR "CRISPR-Cas Systems" OR "Clustered Regularly Interspaced Short Palindromic Repeats" OR "CRISPR-Associated Proteins" OR Clustered Regularly Interspaced Short Palindromic Repeats [Mesh] OR CRISPR-Associated Proteins [Mesh] OR CRISPR-cas Systems [Mesh] | AND | myotonic dystrophy OR "myotonic dystrophy" OR myotonic dystrophy [Mesh] |
|  | 9 |  |  | dystrophy OR muscular dystrophy [Mesh] OR "neuromuscular disease" OR "neuromuscular disorder" |
|  | 10 |  |  | trinucleotide repeat expansion OR "trinucleotide repeat expansion"  OR trinucleotide repeat expansion [Mesh] |
|  | 11 |  |  | Huntington's disease OR "Huntington's disease" OR Huntington disease [Mesh] |
|  | 12 |  |  | spinocerebellar ataxia* OR "spinocerebellar ataxia" OR spinocerebellar ataxias [Mesh] |
|  | 13 |  |  | Friedreich* ataxia* OR "Friedreich ataxia" OR Friedreich ataxia [Mesh] |
|  | 14 |  |  | fragile X syndrome OR "fragile X syndrome" OR fragile X syndrome [Mesh] |
| EMBASE | 15 | gene editing.mp. OR gene editing/ | AND | myotonic dystrophy.mp. OR myotonic dystrophy |
|  | 16 |  |  | dystrophy/ OR dystrophy.mp. OR neuromuscular.mp. OR neuromuscular disease |
|  | 17 |  |  | Huntington chorea/ OR huntington disease.mp. OR trinucleotide repeat/ OR trinucleotide repeat disorder.mp. OR fragile X syndrome/ OR fragile X syndrome.mp. |
|  | 18 |  |  | friedreich ataxia.mp. OR Friedreich ataxia/ OR spinocerebellar ataxia.mp. OR spinocerebellar degeneration/ |
|  | 19 | CRISPR Cas system/ OR clustered regularly interspaced short palindromic repeat/ OR CRISPR cas9.mp. | AND | myotonic dystrophy.mp. OR myotonic dystrophy |
|  | 20 |  |  | dystrophy/ OR dystrophy.mp. OR neuromuscular.mp. OR neuromuscular disease |
|  | 21 |  |  | Huntington chorea/ OR huntington disease.mp. OR trinucleotide repeat/ OR trinucleotide repeat disorder.mp. OR fragile X syndrome/ OR fragile X syndrome.mp. |
|  | 22 |  |  | friedreich ataxia.mp. OR Friedreich ataxia/ OR spinocerebellar ataxia.mp. OR spinocerebellar degeneration/ |
